# Drug Addiction Endophenotypes: Impulsive Versus Sensation-Seeking Personality Traits

**DOI:** 10.1016/j.biopsych.2010.06.015

**Published:** 2010-10-15

**Authors:** Karen D. Ersche, Abigail J. Turton, Shachi Pradhan, Edward T. Bullmore, Trevor W. Robbins

**Affiliations:** a Department of Psychiatry, University of Cambridge, Cambridge, United Kingdom; b Department of Experimental Psychology, University of Cambridge, Cambridge, United Kingdom; c Department of Behavioural and Clinical Neuroscience Institute, University of Cambridge, Cambridge, United Kingdom; d GlaxoSmithKline, Clinical Unit Cambridge, Cambridge, United Kingdom

**Keywords:** BIS-11, endophenotypes, impulsivity, sensation-seeking, substance dependence, vulnerability marker

## Abstract

**Background:**

Genetic factors have been implicated in the development of substance abuse disorders, but the role of pre-existing vulnerability in addiction is still poorly understood. Personality traits of impulsivity and sensation-seeking are highly prevalent in chronic drug users and have been linked with an increased risk for substance abuse. However, it has not been clear whether these personality traits are a cause or an effect of stimulant drug dependence.

**Method:**

We compared self-reported levels of impulsivity and sensation-seeking between 30 sibling pairs of stimulant-dependent individuals and their biological brothers/sisters who did not have a significant drug-taking history and 30 unrelated, nondrug-taking control volunteers.

**Results:**

Siblings of chronic stimulant users reported significantly higher levels of trait-impulsivity than control volunteers but did not differ from control volunteers with regard to sensation-seeking traits. Stimulant-dependent individuals reported significantly higher levels of impulsivity and sensation-seeking compared with both their siblings and control volunteers.

**Conclusions:**

These data indicate that impulsivity is a behavioral endophenotype mediating risk for stimulant dependence that may be exacerbated by chronic drug exposure, whereas abnormal sensation-seeking is more likely to be an effect of stimulant drug abuse.

Substance dependence has been associated with a variety of psychopathological disturbances, but it is still a matter of debate to what extent these are the result of chronic drug abuse or a predisposing risk factor. Personality traits of impulsivity and sensation-seeking are highly prevalent in substance-dependent individuals and have both been discussed as determinants and consequences of substance abuse (1,2). Impulsivity has been defined as a loss of inhibitory control over the response to rewarding or distracting stimuli and is frequently assessed by self-report measures such as the Barratt Impulsiveness Scale, Version 11 (BIS-11) (3). Preclinical models of drug addiction indicate that impaired inhibitory control increases the risk of drug-taking behaviors, paving the way for the development of the out-of-control drug-seeking pattern seen in drug addiction (4). Sensation-seeking has been defined as a need to seek intense sensations along with the willingness to take risks for the sake of having such experiences. It can be assessed by self-report using the Zuckerman Sensation-Seeking Scale Form V (SSS-V) (5). Underresponsiveness to natural rewards and the need for greater stimulation have been suggested to motivate drug taking (6).

Drug addiction runs in families and relatives of drug-dependent individuals have an eightfold increased risk of developing substance abuse disorders compared with the general population (7). If impulsivity and sensation-seeking are endophenotypes or vulnerability markers of addiction, high levels of both personality traits would not only be observed in drug-dependent individuals but also in their full brothers/sisters who do not have a significant drug-taking history. We therefore assessed impulsivity and sensation-seeking using standardized questionnaires in 30 sibling pairs and 30 healthy, nondrug-taking control volunteers.

## Methods and Materials

### Participants

The protocol was approved by the Cambridge Research Ethics Committee and written informed consent was obtained from all volunteers. Participants were recruited upon referral from treatment services and through media advertisements. One drug user withdrew consent; consequently, her data were not included in the study. All participants underwent a screening process, which included a semistructured interview to ascertain their history of drug use, their general and mental health, and demographic characteristics. Sibling pairs were included if three conditions were met: 1) same biological parents, 2) one sibling satisfied the *Diagnostic and Statistical Manual, Fourth Edition, Text Revision* (DSM-IV-TR) criteria for cocaine or amphetamine dependence, and 3) the other sibling had no personal history of substance dependence (except nicotine). Exclusion criteria, which applied for all groups, were a lifetime history of a psychotic disorder, a history of a neurological disorder, or a traumatic head injury. Participants had to be 18 to 55 years old and able to read and write in English.

All drug users met the DSM-IV-TR criteria for stimulant dependence (93% cocaine and 7% amphetamines). On average, they had been using stimulants for 16.3 years (±7.6 SD), starting at the age of 16.5 years (±3.0 SD). Almost half of the drug user sample also met criteria for dependence on another substance (45% opiates, 31% alcohol, and 14% cannabis). One drug user was positive for the human immunodeficiency virus. The drug-taking experiences and use of alcohol were notably low in the sibling and the control groups, as reflected by low scores on the Drug Abuse Screening Test (8) and the Alcohol Use Disorders Identification Test (9), shown in Table 1.

### Procedures

All participants were screened for any other current Axis I psychiatric disorder using the Structured Clinical Interview for the DSM-IV-TR Axis I Disorders (SCID) (10) and completed the National Adult Reading Test (11) as an estimate of verbal IQ. The Beck Depression Inventory, Second Edition (BDI-II) (12) was used to record depressive mood. Trait-impulsivity was assessed using the BIS-11 (3), which consists of three subscales: 1) attentional impulsiveness (inattention and cognitive instability), 2) motor impulsiveness (spontaneous actions), and 3) nonplanning impulsiveness (lack of forethought). Sensation-seeking was assessed using the SSS-V (5), which consists of four subscales: 1) thrill and adventure-seeking (a desire to participate in dangerous activities), 2) experience-seeking (search for new experiences in a nonconformist manner), 3) disinhibition (interest in socially and sexually disinhibited activities), and 4) boredom susceptibility (intolerance of routines and repetitiveness).

### Statistical Analysis

Data were analyzed using the Statistical Package for Social Sciences, version 13 (SPSS, Inc., Chicago, Illinois). One-way analyses of variance were used to explore group differences in demographics, and chi-square or Fisher's exact tests were used for the analysis of categorical data.

Univariate analysis of covariance models were fit to the BIS-11 and the SSS-V total scores, whereas the subscales of these two instruments were analyzed using separate multivariate analysis of covariance models. Gender was included as a covariate in all analyses and the BDI-II was included if correlated with the dependent variable. For post hoc comparisons, the Dunn-Sidak correction was applied. Pearson correlations were estimated where appropriate. All tests were two-tailed and a significance level of .05 was assumed.

## Results

Demographic data on the three groups are shown in Table 1. Drug users reported significantly higher levels of dysphoric mood compared with their siblings and with control volunteers (both *p* < .001). Correlations between the BDI-II and both the BIS-11 and SSS-V total scores were nonsignificant in each of the groups separately. For the subscales, the BDI-II was significantly correlated with BIS-11 nonplanning (*r* = .43, *p* < .05) and the SSS-V thrill/adventure-seeking subscale (*r* = .52, *p* < .001) in the drug user group. Therefore, the BDI-II score was included as a covariate in the further analysis of these traits.

**Figure 1:**
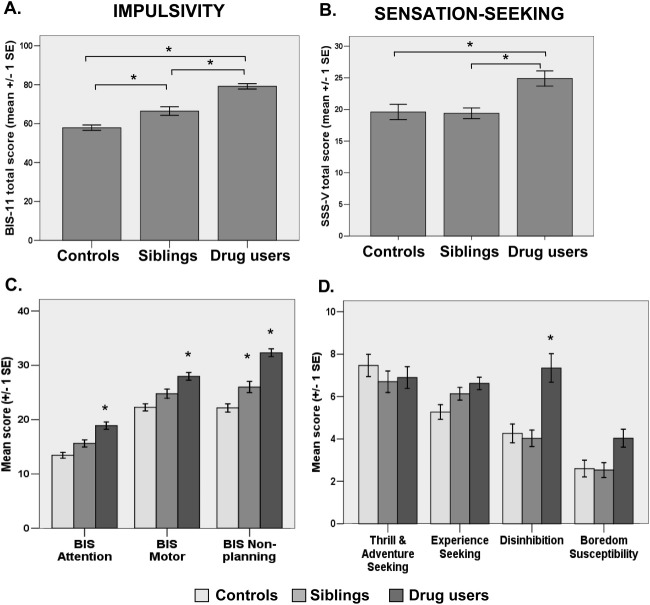
Comparisons between stimulant-dependent individuals, their full biological bothers/sisters, and unrelated healthy control volunteers with regard to **(A)** impulsive personality traits, as reflected by the Barratt Impulsiveness Scale, Version 11 total score, and **(B)** sensation-seeking personality traits, as reflected by the Sensation-Seeking Scale Form V total score. **(C)** The group differences in impulsivity are also reflected on the Barratt Impulsiveness Scale, Version 11 subscales for attentional, motor, and nonplanning impulsivity, and **(D)** on the Sensation-Seeking Scale Form V subscales for thrill and adventure-seeking, experience-seeking, disinhibition, and boredom susceptibility (note: the asterisks indicate the significant differences). BIS-11, Barratt Impulsiveness Scale, Version 11; SSS-V, Sensation-Seeking Scale Form V.

The groups differed significantly on self-reported impulsivity [*F*(2,85) = 38.70, *p* < .001], as shown in Figure 1A. Both drug users (*p* < .001) and siblings (*p* = .004) reported significantly higher levels of trait-impulsivity than control volunteers. Drug users were also more impulsive than their siblings (*p* < .001). The significant group differences were also reflected on all three BIS-11 subscales (Wilk's lambda = .72, *F* = 4.46, *p* < .001). Post hoc tests confirmed that, compared with control volunteers, the high levels of impulsivity in drug users were significant on all the subscales (all *p* ≤ .001). The siblings, however, only differed from control volunteers (*p* = .024) and from the drug users (*p* = .012) on the BIS-11 nonplanning subscale.

Figure 1B shows that the groups also differed significantly on sensation-seeking, as reflected by the SSS-V total score [*F*(2,85) = 5.62, *p* = .005]. Drug users reported significantly higher levels of sensation-seeking compared with both their siblings (*p* = .025) and with control volunteers (*p* = .008). No difference in sensation-seeking was found between control volunteers and the siblings (*p* = .988). The overall analysis of the four SSS-V subscales was significant (Wilk's lambda = .71, *F* = 3.87, *p* < .001), but post hoc testing showed that the groups only differed significantly on the disinhibition subscale [*F*(2,84) = 5.79, *p* = .004]; drug users reported a greater interest in disinhibited activities compared with both the control volunteers (*p* = .007) and their siblings (*p* = .007). For further results, see Supplement 1.

## Discussion

Trait-impulsivity was not only increased in the drug users but also in their siblings, indicating that it could be an endophenotype or vulnerability trait predisposing to development of stimulant dependence. This is intriguing because BIS-11 scores have been associated with neurochemical and structural variations in brain areas that are implicated in the pathophysiology of drug addiction: thus, high BIS-11 scores have been linked with reduced striatal dopamine receptor availability (13) and reduced gray matter volume in the orbitofrontal cortex (14). Drug addiction involves neuroadaptive changes within large-scale striato-thalamo-orbitofrontal networks implicated in the processing of natural rewards and the regulation of behavior (15). Failures in self-regulation are reflected by impulsivity, and the observation of increased levels of trait-impulsivity in the siblings of drug users suggests that this mechanism may constitute a biological predisposition for drug dependence. Chronic drug exposure may further exacerbate impulsivity, leading to the particularly high BIS-11 scores in drug-dependent individuals (3), as much as drug abstinence decreases BIS-11 scores (16).

The SSS-V scores were significantly increased in the drug users but not in their siblings, suggesting that sensation-seeking is not an endophenotype for stimulant dependence. The increased SSS-V total score in the drug users was driven by significantly higher scores on the disinhibition subscale, which overlaps with the loss of control component of impulsivity. This may explain why the SSS-V and the BIS-11 were correlated in the stimulant users but not in the other two groups. In light of the preclinical evidence indicating that dopamine receptor levels in the brain reward system decline following chronic drug exposure (17), it is conceivable that sensation-seeking, in particular increased SSS-V disinhibition, is a result of chronic drug exposure. Dopamine receptors in the striatum have been shown to mediate both the reinforcing effects of stimulant drugs (18) and the expression of sensation-seeking traits (19).

Although sensation-seeking does not meet criteria for an endophenotype of stimulant dependence, our findings do not rule out that high levels of sensation-seeking might have preceded stimulant abuse. Indeed, sensation-seeking has been linked with the initiation of substance abuse, but impulsivity, rather than sensation-seeking, has been associated with the development of stimulant dependence (20). It is therefore arguable that relatively low levels of sensation-seeking behavior in the siblings may have protected them from initial exposure to stimulant drugs and therefore counteracted the predisposing effects of high impulsivity on development of stimulant dependence. Further clarification of the potential causes and the chronic effects of drug dependence may have a fundamental impact on the future development of therapeutic and preventative interventions. Here, we have shown that impulsivity is likely to be a heritable risk factor predisposing to the development of stimulant dependence.

## Figures and Tables

**Table 1:** Demographic, Psychological, and Baseline Personality Measures for the Groups of Stimulant-Dependent Individuals, Their Full Brothers/Sisters, and Healthy Control Volunteers

Group	Control Volunteers	Siblings	Drug Users	*F* or χ^2^	df	*p*
*n*	30	30	29			
Age	31.9 (± 9.4)	33.7 (± 8.5)	33.4 (± 8.4)	.45	2,86	.640
Gender (Male:Female)	22:8	16:14	26:3	Fisher exact		.008
Handedness (Right:Left)	25:5	27:3	24:5	Fisher exact		.752
Birth Rank (Youngest, Middle, Eldest Child)	12:9:9	9:13:8	10:10:9	1.31	4	.860
Parents Divorced/Separated^a^	25%	57%	59%	8.91	2	.012
Age When Parents Divorced	9.6 (± 4.8)	7.5 (± 5.3)	8.4 (± 4.2)	.52	2,42	.601
Same Upbringing Until Age 15^b^	—	87%	93%	Fisher exact		.671
Verbal Intelligence (NART)	111.0 (± 7.0)	111.3 (± 6.9)	109.0 (± 7.2)	.67	2,80	.513
Years of Education	12.7 (± 1.8)	12.2 (± 1.9)	11.70 (± 1.5)	2.48	2,86	.089
Depressive Mood (BDI-II)	1.7 (± 2.2)	4.9 (± 7.2)	18.41 (± 11.9)	35.34	2,86	<.001
Cigarettes Per Day	10.0 (± 0)^*n* = 2^	10.7 (± 8.9)^*n* = 19^	15.7 (± 8.0)^*n* = 26^	2.15	2,44	.129
Alcohol Screen (AUDIT Score)	3.7 (± 2.7)	2.9 (± 3.0)	14.4 (± 12.1)	22.70	2,86	<.001
Drug Screen (DAST-20 Score)	.0 (± 0)	.4 (± 1.0)	—	5.47	1,60	.023

Scoring: BDI-II (0–13 minimal, 14–19 mild, 20–28 moderate, 29–63 severe); AUDIT (0–7 safe drinking, 8 > harmful drinking); DAST-20 (0 none, 1–5 low severity, 6–10 intermediate severity, >11 substantial severity). AUDIT, Alcohol Use Disorders Identification Test; BDI-II, The Beck Depression Inventory, Second Edition; DAST-20, Drug Abuse Screening Test; NART, National Adult Reading Test.

a The different percentages in sibling and drug users are because one drug user withdrew consent.

b The different percentages in siblings and drug users are due to different birth ranks and differences in age at the time of their separation.
