# Supplementary material for: Drug Addiction Endophenotypes: Impulsive Versus Sensation-Seeking Personality Traits
Source: Biol Psychiatry. 2010 Oct 15;68(8):770–3. doi: 10.1016/j.biopsych.2010.06.015 (PMC3485555; doi:10.1016/j.biopsych.2010.06.015)
Supplement: Supplement 1 [file mmc1.pdf]

# Supplemental Information

Impulsivity and sensation-seeking were significantly correlated in drug users ($r = 0.54$, $P < 0.01$) but not in controls ($r = 0.34$, $P > 0.05$) or in their siblings ($r = 0.12$, $P > 0.05$). BIS-11 and SSS-V total scores were not correlated with the duration of stimulant abuse or the age of stimulant abuse onset.

We further investigated differences in impulsivity and sensation-seeking traits between smokers (n = 21) and non-smokers (n = 39) in the siblings and the unrelated healthy volunteers; drug users were excluded from this subgroup analysis since almost all of them were smokers. Smokers reported significantly greater levels of sensation-seeking compared with non-smokers ($F_{1,56} = 5.65$, $P = 0.021$). In contrast to the stimulant drug users, tobacco smoking was associated with increased scores on the experience-seeking subscale ($F_{1,56} = 10.20$, $P = 0.002$) rather than the disinhibition, suggesting that sensation-seeking may be qualitatively different between tobacco smokers depending on whether they are also dependent on stimulant drugs .

Thus, no group differences between smokers and non-smokers were observed on the SSS-subscales of thrill-seeking ($F_{1,56} = 2.20$, $P = 0.144$), disinhibition ($F_{1,56} = 1.78$, $P = 0.188$), and boredom susceptibility ($F_{1,56} = 0.32$, $P = 0.859$). We further observed a trend to greater impulsivity on the BIS-11 in smokers ($F_{1,56} = 3.52$, $P = 0.066$), but this was not formally significant or reflected in differences on the BIS-11 subscales

High levels of impulsivity and sensation-seeking were consistent across stimulant drug users, whether or not they were also dependent on opiates, as no difference on the BIS-11 ($F_{1,27}$ = 0.07, *P* = 0.800) or SSS-V ($F_{1,27}$ = 0.20, *P* = 0.662) scales were observed between stimulant users with (n = 13) and without (n = 16) co-morbid opiate dependence. There were also no differences between these subgroups on the subscales of both questionnaires.
